# Gender-Specific Impacts of Apnea, Age, and BMI on Parasympathetic Nerve Dysfunction during Sleep in Patients with Obstructive Sleep Apnea

**DOI:** 10.1371/journal.pone.0092808

**Published:** 2014-03-25

**Authors:** Kazuhiro Yamaguchi, Yuji Inoue, Noboru Ohki, Natsumi Satoya, Fukumi Inoue, Yoshiko Maeda, Haruki Sekiguchi, Mayumi Suzuki, Takao Tsuji, Kazutetsu Aoshiba, Atsushi Nagai

**Affiliations:** 1 Comprehensive Medical Center of Sleep Disorders, Aoyama Hospital, Tokyo Women’s Medical University, Minato-ku, Tokyo, Japan; 2 NoruPro Light Systems Incorporation, Kokubunji-shi, Tokyo, Japan; 3 Department of Urology, Aoyama Hospital, Tokyo Women’s Medical University, Minato-ku, Tokyo, Japan; 4 Department of Cardiology, Aoyama Hospital, Tokyo Women’s Medical University, Minato-ku, Tokyo, Japan; 5 Department of Respiratory Medicine, Tokyo Medical University Ibaraki Medical Center, Inashiki, Ibaraki, Japan; 6 The First Department of Medicine, Tokyo Women’s Medical University, Shinjuku-ku, Tokyo, Japan; University of Barcelona, Faculty of Biology, Spain

## Abstract

**Background:**

The gender-specific influences of various confounding factors, including apnea, age, BMI, and cigarette consumption, on the function of the parasympathetic nerve system (PNS) during sleep in OSA patients has never been investigated.

**Methods:**

One hundred ninety-seven males and 63 females with OSA were subjected to full PSG examinations including assessment of R-R intervals (RRIs) during an overnight ECG. The PNS-derived modulatory effect on the RRIs and the variability of this effect were quantified during REM and NREM using instantaneous time-frequency analysis with complex demodulation. The spectral domain with the maximum instantaneous amplitude in the high-frequency band between 0.15 and 0.4 Hz was defined as the main HF peak and used as a surrogate marker of PNS discharge. Based on density-spectrum-array maps of the main HF peaks (HF-DSA map), shifts in the central frequency of the main HF peak over time were continuously observed. When the main HF peaks on the HF-DSA maps maintained the same central frequency for more than 20 sec or 5 min, the PNS functions were considered to be “stable” or “very stable”, respectively.

**Results:**

Apneas enhanced PNS-derived cardiac-modulation during REM in males, but more importantly, they made PNS-function unstable during both REM and NREM in males and during NREM in females. Aging blunted the PNS-derived cardiac-modulation during both REM and NREM regardless of gender, but aging had no impact on the stability of PNS-function. BMI blunted PNS-eliciting cardiac-modulation during REM in males and during NREM in both males and females. BMI made the PNS unstable during REM in females. Neither height nor cigarette consumption influenced any PNS-related parameter.

**Conclusions:**

The PNS-derived cardiac-modulation was generally inhibited by aging and obesity, in which the effect of obesity was gender-specific. The PNS instability at nighttime was mainly induced by apneas but by obesity particularly during REM in females.

## Introduction

Although the dysfunction of the parasympathetic nerve system has been considered to be implicated in the pathogenesis of a variety of cardiovascular comorbidities in patients with obstructive sleep apnea (OSA), the results reported for the parasympathetic nerve function in OSA patients have highly been inconsistent [1]–[3]. We have demonstrated that these inconsistencies are ascribed to methodological problems [4]; i.e., they were estimated based on the power-spectrum analysis on heart rate variability (HRV) elicited by the efferent parasympathetic nerve discharge traveling to the cardiac sinus node. Although the power-spectrum analysis is a useful method while estimating the parasympathetic nerve function in subjects with no apnea and hypopnea [5], [6], it is difficult to apply this method to OSA patients having significant episodes of apneas and hypopneas during sleep. The time resolution of the classical power-spectrum analysis is low and requires at least 100 heat beats (corresponding to approximately 2 min) to obtain the data necessary for a definitive analysis of the frequency domains contained in the R-R heartbeat interval, which is representative of HRV [5], [6]. Within 2 min, a subject with severe apneas will experience difficulties with respiration influencing the parasympathetic nerve function. Therefore, the change in the parasympathetic nerve function related to the distorted respiration around the time points of apneic events may be missed when using the classical methods. To overcome these faults involved in the classical methods, we have recently developed the novel method enabling us to measure a change in parasympathetic nerve discharge to the cardiac sinus node in a practically continuous manner [4]. We termed this novel method “instantaneous time-frequency analysis”, which was established as the basis of the complex demodulation method [7], [8]. The complex demodulation method allows us to measure a transitional change in instantaneous amplitude of a target frequency domain from a short-time tracing of seven heart beats (corresponding to 6.7 sec), indicating that the complex demodulation method can detect the change in parasympathetic nerve discharges during apneic episodes lasting 10 sec or more. In the previous paper, we confirmed that the complex demodulation method was a promising tool for the quantification of parasympathetic nerve discharges to the cardiac sinus node during the night in OSA patients with morbid apneas [4]. In addition, we found that parasympathetic nerve function during REM and NREM sleep in OSA patients is conspicuously disturbed and that this disturbance is successfully restored with CPAP treatment [4]. However, in our previous paper, we did not exclude the effects of various confounding factors, such as age, gender, body mass index (BMI), and cigarette consumption, on modification of parasympathetic nerve dysfunction in OSA patients. Age, gender, BMI, and cigarette consumption were demonstrated to have a significant impact on the parasympathetic nerve function in subjects with normal respiration [9]–[22]. It may be clinically important, therefore, to account for the effects of these confounding factors when attempting to precisely estimate impairments in parasympathetic nerve function. However, to the best of our knowledge, no comprehensive studies of the influence of various confounding factors on parasympathetic nerve dysfunction in patients with apneic episodes have been conducted. Several decades ago, Hrushesky et al. [9] and Shannon et al. [10] analyzed the parasympathetic-nerve-related modulatory effects on the cardiac sinus node and revealed that these effects are substantially inhibited by aging. However, these investigations were performed in awake subjects with normal respiration and not in sleeping subjects with morbid apneas. Based on the time-domain analysis of HRV, Song et al. [23] studied the effect of age on HRV in male subjects with OSA, demonstrating that aging would play an important role in blunting the parasympathetic-nerve-eliciting modulatory effect on the cardiac sinus node in these subjects, as well. However, they did not measure the HRV-related time-domain variables in females with OSA. Based on these historical backgrounds, the present study was undertaken to clarify the gender-specific impacts of apnea, age, anthropometric factors (height, body weight, and BMI), and life-long cigarette consumption on the parasympathetic nerve dysfunction during REM and NREM sleep in patients with OSA using the complex demodulation method.

## Materials and Methods

### Ethics statement

All participants provided written informed consent that their data would be used for clinical research and agreed to the inclusion of their data in a database that would be used for various research programs. Our research protocol was approved by the Human Ethics Committee of the Tokyo Women’s Medical University.

### Study population

Eligible participants were selected from patients who were referred to our Comprehensive Medical Center of Sleep Disorders between 2010 and 2012 with sleep-related complaints (n = 826, males: 603, females: 223). Each subject was required to complete questionnaires covering age, height, body weight, life-long cigarette consumption, drinking, snoring, nocturnal urination, breathing pattern (i.e., through the nose or the mouth), types of dreams, depressive feelings, restless legs symptoms, gastroesophageal reflux, nasal congestion, and medical histories regarding comorbidities and medications. Subjects also completed the Epworth Sleepiness Scale and the Athens Insomnia Scale. The subject then underwent full overnight polysomnography (PSG) examinations at the sleep laboratory of the Center. Based on the information recorded in the questionnaires, the results of the physical examinations, chest X-rays, electrocardiograms, blood examinations, and PSG data, the following subjects were excluded from the analysis; (1) the subjects who refused to participate in the study or those who were joining in other studies concurrently advanced in the Center (n = 643, males: 392, females: 251). (2) the subjects who had no abnormal respiration during sleep (overall apnea-hypopnea index (AHI) < 5 events/hr) (n = 18, males: 13, females: 5), (3) the subjects with central sleep apneas (overall AHI ≥ 5 events/hr in which central sleep apnea predominated) (n = 16, males: 7, females: 9), (4) the subjects with any pathological condition, including malignancy in any organ, severe heart failure, heart attack or stroke, renal failure requiring dialysis, or impaired cognitive function (n = 42, males: 31, females: 11), and (5) the subjects who were taking β agonists, β antagonists or anti-cholinergic agents, and those with atrial fibrillations or artificial cardiac rhythms generated by pacemakers (n = 107, males: 78, females: 29). Thus, 260 participants with OSA (overall AHI ≥ 5 events/hr) finally met the inclusion criteria (197 males and 63 females) (Table 1). The age distributions of the male and female participants ranged from 31 to 84 years old and from 41 to 80 years old, respectively, and the BMIs ranged from 17.7 to 42.7 kg/m^2^ and 15.9 to 39.1 kg/m^2^ in the males and females, respectively. The life-long cigarette consumption of the females averaged 4.0 pack-years, and the average for the males was much higher (21.6 pack-years). Among the males, 31% had never smoked, and 71% of the females had never smoked.

**Table 1:** Basic characteristics of male and female participants with OSA.

	Males (n = 197)	Females (n = 63)
Age (years)	61.0±10.8 (31, 84)	63.5±10.4 (41, 80)
Height (m)	1.68±0.06 (1.47, 1.83)	1.56±0.06^*^ (1.45, 1.79)
Body weight (kg)	72.9±12.2 (48.0, 125.0)	58.5±11.7^*^ (34.7, 88.0)
Body mass index (kg/m^2^)	25.7±3.8 (17.7, 42.7)	24.1±4.9^*^ (15.9, 39.1)
Life-long cigarette consumption (pack-years)	21.6±23.6 (0, 122.5)	4.0±7.4^*^ (0, 23.0)
ESS	8.2±4.6 (0, 18.0)	7.0±4.4^*^ (1, 15.0)
Frequency of nocturnal urination (numbers/night)	1.4±1.2 (0, 5.0)	0.7±0.81^*^ (0, 3.0)

ESS: Epworth Sleepiness Scale. Among the males, 31% had never smoked, and 71% of the females had never smoked. The ranges of each parameter are indicated in parentheses (minimum, maximum). ^*^ p<0.04 compared to the values obtained for males.

### Instantaneous time-frequency analysis for estimating parasympathetic nerve function

**Figure 1:**
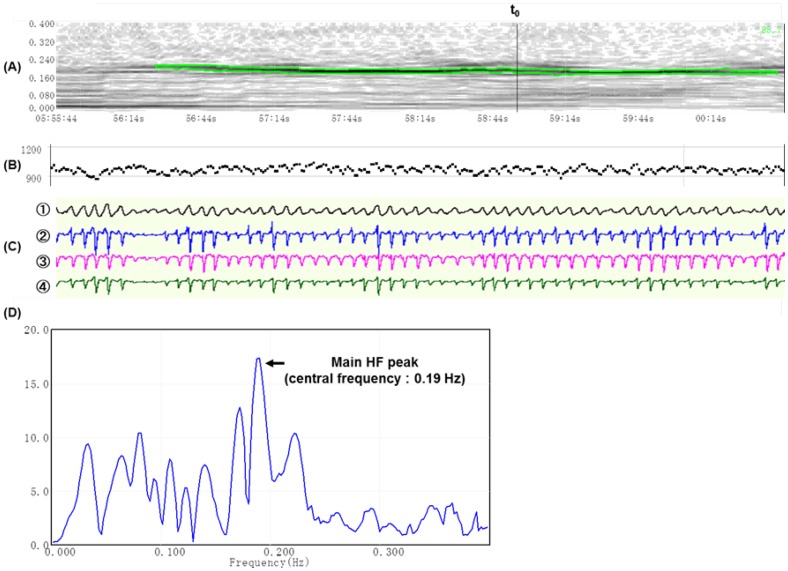
HF-DSA map constructed via instantaneous time-frequency analysis. These data were collected from a male subject with severe OSA who was 54 years old with a height of 181/m^2^, and an overall AHI of 57.3/hr. (A): HF-DSA map during NREM with hypopneas (observation time: 5 min). Vertical axis: frequency (Hz). Horizontal axis: time (min : sec). The green band with the black line at the center denotes the stable main HF peak with a central frequency of 0.19 Hz. (B): RRI (msec). (C): PSG data: 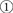 oral airflow measured with thermocouples, 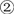 nasal airflow by nasal air pressure, 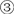 and 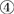 respiration-induced thoracic and abdominal motions recorded by piezoelectric belt sensors, respectively. (D): Frequency spectra at time t_0_ on the HF-DSA map. Vertical axis: instantaneous amplitudes of HF peaks (msec). Horizontal axis: frequency (Hz).

To evaluate parasympathetic nerve function during REM and NREM sleep in patients with OSA, we performed instantaneous time-frequency analyses on HRVs as represented by the R-R intervals using a complex demodulation method [4], [7], [8]. The details of the complex demodulation method and its application to OSA patients with morbid apneas are provided in our previous paper [4]. Briefly, the complex demodulation method involves calculating the instantaneous amplitudes of all spectral domains in the high frequency (HF) region between 0.15 and 0.40 Hz from the R-R interval data at specific time points. From the HF domains, the main HF with the maximum instantaneous amplitude is identified and assumed to reflect the efferent parasympathetic nerve discharges transmitted to the cardiac sinus node at that specific time point concerned [24]. This complex demodulation method enabled us to construct maps that allow for investigation of the time-dependent variations in frequency of the main HF peak in a practically continuous manner. We termed these maps of the main HF peaks “density-spectrum-array maps” (HF-DSA maps) (Fig. 1). The average value of the instantaneous amplitudes of the respective main HF peaks obtained for the entire duration of REM or NREM sleep was defined as the “average HF amplitude” and used as the parameter expressing the average extent of parasympathetic-nerve-elicited modulation of the cardiac sinus node in either phase of sleep [25]. When the main HF peak maintained the same central frequency for least 20 sec without any disruption in the HF-DSA map, the parasympathetic nerve function was taken to be stable. The duration in which the main HF peak was stable over 20 sec was defined as the HF_20sec_. The ratios of the HF_20sec_ to total NREM and REM times were denoted as the %HF_20sec_ at NREM and the %HF_20sec_ at REM, respectively, and these measures were used as practical parameters to judge the stabilities of parasympathetic nerve function during these sleep stages. Similarly, when the main HF peak maintained the same central frequency for more than 5 min (HF_5min_), the parasympathetic nerve function was assumed to be very stable, and the very stable conditions were assessed with ratios of HF_5min_ to total NREM or REM times (%HF_5min_). As such, the newly developed instantaneous time-frequency analysis as the basis of the complex demodulation method enabled us to quantitatively estimate the magnitude as well as the stability of the parasympathetic nerve modulation on the cardiac sinus node.

### Covariate analyses and statistics

In addition to AHI, we used age, height, body weight, BMI, and life-long cigarette consumption as confounding, explanatory variable (independent variable) that could modify the parasympathetic nerve dysfunction during sleep in OSA patients. To determine the independent contribution of each explanatory variable to the parasympathetic-nerve-evoked modulation effect on HRV and to the stability of the parasympathetic nerve function, we calculated the partial correlation coefficient between each explanatory variable and each of the following objective variables (dependent variables): average HF amplitude, %HF_20sec_, and %HF_5min_ in NREM and REM times. However, in these calculations, the partial correlation coefficients for body weight and BMI were unstable due to the strong correlation between the two, i.e., the multicolinearity between body weight and BMI. Therefore, we excluded body weight from the partial correlation coefficient estimations and assumed that the effects of body weight on each of the parasympathetic-nerve-related parameters would be included in those of BMI. Because the extent of the contribution of any confounding factor to parasympathetic nerve function was expected to differ significantly between the males and females, a gender-specific partial correlation coefficient was estimated for each confounding factor.

All calculations were performed using the IBM SPSS package (Version 21.0, IBM SPSS Inc.; NY, USA). Unless otherwise specified, the values are expressed as the means ± the SD. P-values lower than 0.05 were deemed statistically significant.

## Results

### Overall characteristics of REM- and NREM-associated parasympathetic nerve function in each gender (Table 2)

Although there were no great differences in age and BMI distributions between males and females with OSA (Table 1), the AHI values in REM and NREM differed significantly between the genders. In the female OSA, the AHI value in REM sleep was larger, while that in NREM sleep was smaller, than those observed for the male OSA (both: p<0.02). The AHI during REM was larger than that during NREM in the female OSA (p<0.05). On the contrary, the AHI during REM was smaller than that during NREM in the male OSA (p<0.05).

In each gender, the average HF amplitude during NREM sleep was larger and the %HF_20sec_ and %HF_5min_ during NREM sleep were much longer compared to REM sleep (p<0.05 for each). Although no gender-related differences in average HF amplitude or %HF_5min_ were observed during REM sleep, the %HF_20sec_ during REM sleep was longer in the females (p<0.02). Meanwhile, the %HF_20sec_ and %HF_5min_ during NREM sleep were much longer in the females (p<0.02). No difference in average HF amplitude between genders was observed during NREM sleep.

### Influence of confounding factors on parasympathetic nerve function during REM and NREM sleep (Table 3)

**Figure 2:**
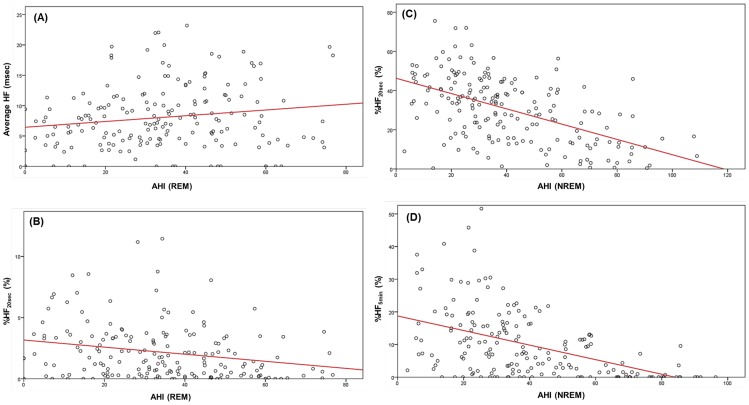
Impacts of AHI on parasympathetic nerve function during REM and NREM in males. Vertical axis: average value of instantaneous amplitudes of the main HF peaks (average HF) in (A), %HF_20sec_ in (B) and (C), and %HF_5min_ in (D). Abscissa: AHI in REM or NREM. Red line: regression line determined with least-squares minimization. (A): Effect of AHI on the average HF in REM. The partial correlation coefficient between AHI and average HF was 0.210 (p = 0.005). (B): Effect of AHI on the stability of parasympathetic nerve function (%HF_20sec_) in REM. The partial correlation coefficient between AHI and %HF_20sec_ was –0.177 (p = 0.017). (C): Effect of AHI on %HF_20sec_ in NREM. The partial correlation coefficient between AHI and %HF_20sec_ was –0.518 (p = 0.000). (D): Effect of AHI on %HF_5min_ in NREM. The partial correlation coefficient between AHI and %HF_5min_ was –0.461 (p = 0.000).

**Figure 3:**
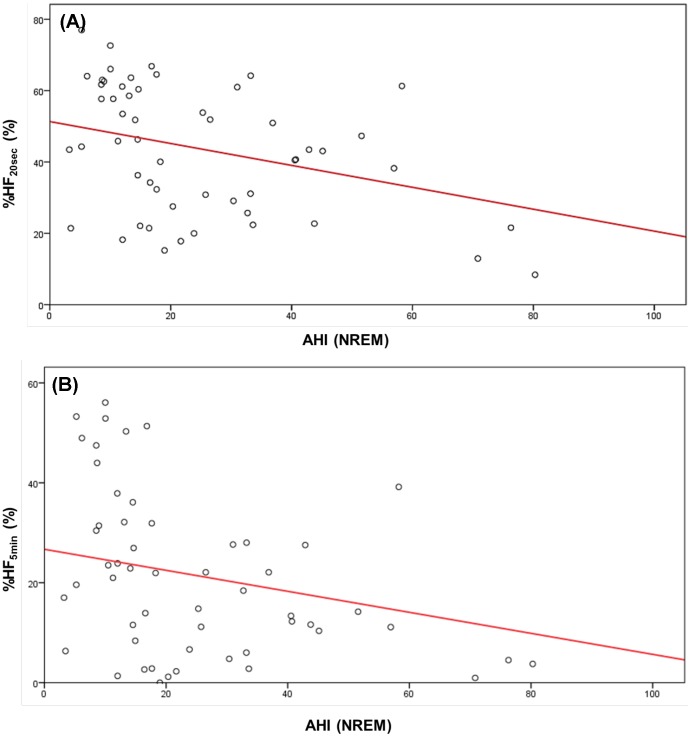
Impacts of AHI on the stability of parasympathetic nerve function during NREM in females. (A): Effect of AHI on %HF_20sec_. The partial correlation coefficient between AHI and %HF_20sec_ was –0.407 (p = 0.003). Red line: regression line. (B): Effect of AHI on %HF_5min_. The partial correlation coefficient between AHI and %HF_5min_ was –0.268 (p = 0.040). Red line: regression line.

Covariate analyses of the partial correlation coefficients demonstrated that, in the males, the AHI augmented the average HF amplitude during REM sleep (p = 0.005), decreased the %HF_20sec_ in both sleep stages (p = 0.017 for REM, p = 0.000 for NREM), and decreased the %HF_5min_ during NREM sleep (p = 0.000) (Fig. 2). In contrast, in the females, AHI had little impact on average HF amplitude but decreased the %HF_20sec_ and %HF_5min_ during NREM sleep (p = 0.003 for %HF_20sec_, p = 0.040 for %HF_5min_) (Fig. 3).

**Figure 4:**
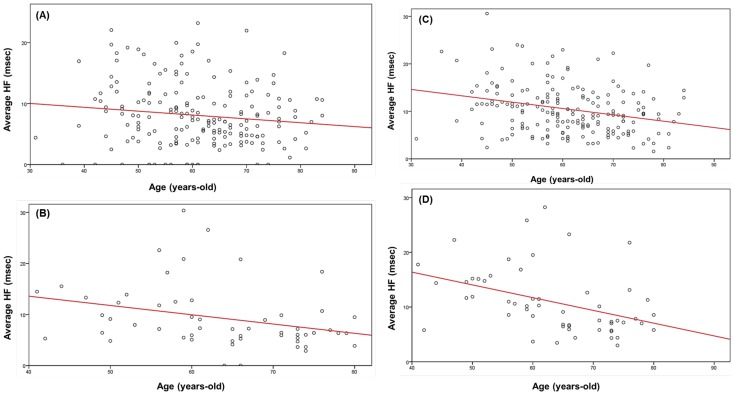
Effects of age on the cardiac modulatory effect of parasympathetic nerve during REM and NREM in both genders. Vertical axis: average value of instantaneous amplitudes of the main HF peaks (average HF). Abscissa: age. Red line: regression line. (A): During periods of REM in males with a partial correlation coefficient between age and average HF of –0.149 (p = 0.045). (B): During REM in females with a partial correlation coefficient between age and average HF of –0.220 (p = 0.048). (C): During NREM in males with a partial correlation coefficient between age and average HF of –0.295 (p = 0.000). (D): During NREM in females with a partial correlation coefficient between age and average HF of –0.357 (p = 0.009).

The average HF amplitudes during REM and NREM sleep in both genders were inversely correlated with age (p = 0.045 for REM in males, p = 0.000 for NREM in males, p = 0.048 for REM in females, p = 0.009 for NREM in females) (Fig. 4). However, age did not affect the %HF_20sec_ or %HF_5min_ of either REM or NREM sleep irrespective of gender.

**Figure 5:**
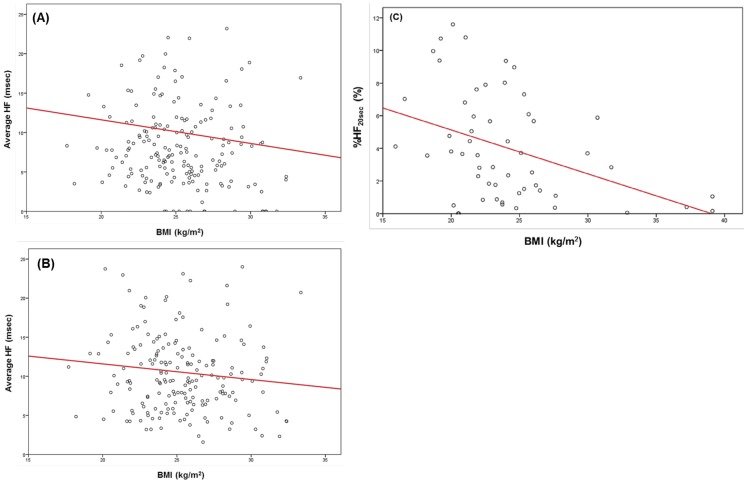
Effects of BMI on the cardiac modulatory effect of the parasympathetic nerve during REM and NREM in males and on the stability of parasympathetic nerve function during REM in females. Vertical axes: average HF in (A) and (B), and %HF_20sec_ in (C). Red line: regression line. (A): The partial correlation coefficient between BMI and average HF during REM sleep in males was –0.187 (p = 0.012). (B): The partial correlation coefficient between BMI and average HF during NREM in males was –0.174 (p = 0.019). (C): The partial correlation coefficient between BMI and %HF_20sec_ during REM in females was –0.369 (p = 0.007).

BMI inversely influenced average HF amplitude during both REM and NREM sleep in the males (p = 0.012 for REM, p = 0.019 for NREM) (Fig. 5), but this effect was restricted to NREM sleep in the females (p = 0.043). However, BMI was inversely correlated with %HF_20sec_ during REM sleep in the females (p = 0.007) (Fig. 5) but not in the males.

Height and life-long cigarette consumption did not influence any parasympathetic nerve parameter in either sleep stages or in either gender.

## Discussion

### Comparison of complex demodulation with classical methods

**Figure 6:**
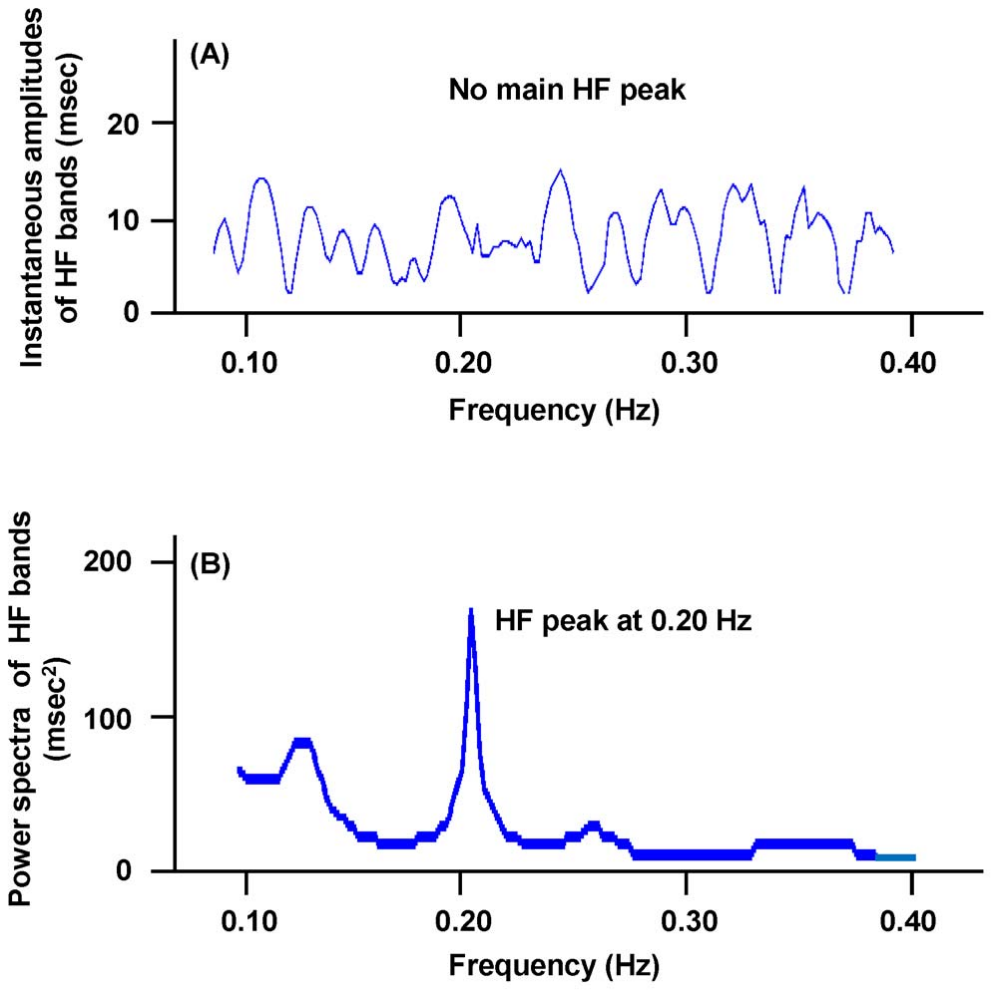
Comparison of frequency spectra obtained by instantaneous time-frequency analysis and maximum entropy method. The data were collected from a male subject with severe OSA who was 52 years old with a BMI of 32.8/m^2^, and an overall AHI of 85.4/hr. (A): Distribution of instantaneous amplitudes of HF bands along frequency axis in HF region decided by instantaneous time-frequency analysis at a certain time point in NREM. Vertical axis: instantaneous amplitudes of HF bands (msec). Horizontal axis: frequency (Hz). Many small HF bands with no main HF peak were identified. (B): Power spectra in HF region decided with maximum entropy method at the same time point as (A). Vertical axis: power spectra of HF bands (msec^2^). Horizontal axis: frequency (Hz). Small HF bands were integrated into single peak with central frequency of 0.20 Hz.

The parasympathetic nerve discharge transmitted to the cardiac sinus node is principally governed by the neural integration of cardiovascular center and respiratory center as well as the reflex from pulmonary stretch receptors [26]. Under a condition with normal respiration, pulmonary stretch receptors are activated by increasing lung volume during inspiration, leading to inhibition of the parasympathetic nerve discharge; i.e., inspiratory gating, while the parasympathetic nerve discharge is augmented by decreasing lung volume during expiration [27], [28]. The parasympathetic-nerve-elicited R-R-interval variation synchronized with inspiratory and expiratory lung volumes generates the respiratory sinus arrhythmia having the main HF peak with a central-frequency at around 0.25 Hz [29], [30]. However, since the integrated neural controls for parasympathetic nerve discharge including the inspiratory gating are disturbed at time points with apneic episodes, the central frequency of the main HF peak under apneic conditions is shifted from 0.25 Hz. Furthermore, it is anticipated that the stability of the parasympathetic nerve function is impeded in OSA patients, leading to a change in central frequency of the main HF peak with time. These facts indicate that the method used for estimating the parasympathetic nerve function in the subject with OSA should have a high resolution for time to follow a dynamically changing respiratory state. The instantaneous time-frequency analysis in terms of complex demodulation adopted in the present study meets the above requirement and enables us to measure a transitional change in instantaneous amplitude of a target frequency domain from a short-time tracing of electrocardiograph recorded for at least 6.7 sec as far as high-frequency domains of R-R intervals are analyzed [4]. However, the classical power-spectrum analysis including fast Fourier transform algorithm or auto-regressive approach requires at least 2 min to obtain the data necessary for an indisputable analysis on frequency domains contained in the R-R intervals. Within 2 min, the subject with severe apneic events certainly encounters the disturbed respiration influencing the parasympathetic nerve function, which is no longer followed by the classical methods. In addition, the classical methods calculate the power spectra integrating the area beneath each frequency component, or summing the square of each amplitude, within a certain range of frequency. Thus, the change in parasympathetic nerve function related to the complicated distortion of respiratory states around time points of apneic events may largely be concealed in the classical methods. As an example, we compared the distribution of HF bands decided by the instantaneous time-frequency analysis and that by the maximum entropy method (one of the power-spectrum analyses) at a certain time point during NREM in a male patient with severe OSA (Fig. 6). We intentionally selected a time period at which the main HF peak was not identified, and only the small HF bands were observed, by the instantaneous time-frequency analysis, suggesting that the parasympathetic-nerve-inducing cardiac modulation would be substantially restricted at this time (Fig. 6-(A)). Since the small HF bands are ascribed to the asynchronous increments and decrements of R-R intervals, they are not directly related to the parasympathetic nerve activity [4]. When calculating the power spectra at the same time point using the maximum entropy method, we observed the single HF peak at 0.20 Hz (Fig. 6-(B)). This single peak was artificially formed by integrating all the small HF bands that were not related to the genuine parasympathetic nerve discharge. Consequently, an erroneous conclusion may be drawn if this single peak is taken as the measure of the parasympathetic nerve discharge under conditions with morbid apneas or hypopneas.

The time-domain analysis has also been used for identifying the autonomic nerve function in subjects with normal respiration [6]. This uses statistical methods to quantify the variation of the standard deviation or the differences between successive R-R intervals. Some of the time-domain variables are considered to mirror the parasympathetic modulation of the heart rate [6], [13], [17], [22], [23]. The time resolution of the time-domain analysis is lower than that of the power-spectrum analysis, because the time-domain analysis requires at least 5 min to obtain the R-R interval data sufficient for a definitive analysis. Nevertheless, we consider that the time-domain analysis is better than the power-spectrum analysis when estimating the parasympathetic nerve function in subjects with OSA, because it uses actual measurements of varied R-R intervals. It should be noted, however, that the stability assessment of the parasympathetic nerve function is not possible when classical methods, including power-spectrum analysis and time-domain analysis, are applied.

### Importance of apneic events for modifying parasympathetic nerve function

We found that the AHI values in REM were much higher, but the AHI values in NREM were much lower, in female OSA than those in male OSA (Table 2), indicating that females and males would be preferably sensitive to REM and NREM regarding the genesis of apneic events, respectively; i.e., the gender-specific difference in the sensitivity to the sleep stage with regard to apnea formation. Meanwhile, we found that the parasympathetic nerve function in both REM and NREM was more stable in females than that in males (Table 2). Although this was supported in part by the fact that the aggravating effect of AHI on the parasympathetic nerve stability was missing during REM in females (Table 3), it is not easy to explain why such a gender-specific difference in the parasympathetic nerve stability occurs in OSA patients. We identified that AHI during NREM or increased BMI during REM would respectively act as the decisive factor for making the parasympathetic nerve system unstable in females (Table 3, Figs. 3 and 5). However, the parasympathetic nerve stability was simply determined by AHI during both REM and NREM in males (Table 3 and Fig. 2). Supporting these findings, our previous study [4] demonstrated that apneic events made the parasympathetic nerve function during both REM and NREM substantially unstable in OSA patients consisting mainly of males and this instability was securely improved when apneas were controlled by CPAP treatment.

**Table 2:** Data regarding sleep stages and parasympathetic nerve function in males and females with OSA.

	Males (n = 197)	Females (n = 63)
Total sleep time (min)	389.8±82.0	397.2±76.8
NREM (min)	340.1±71.6	341.2±63.4
Stage N1 (min)	154.0±70.9	95.0±42.0^*^
Stage N2 (min)	173.9±72.1	210.0±66.9^*^
Stage N3 (min)	13.4±18.2	35.8±24.9^*^
REM (min)	48.5±25.6	55.7±25.4
*REM phase*		
AHI (events/hr)	34.1±18.3	41.3±24.2^*^
Minimum SpO_2_ (%)	80.9±10.4	83.1±8.4
Hear rate (/min)	62.4±9.4	62.3±6.4
HF (msec)	8.1±5.2	9.3±6.2
%HF_20sec_ (%)	2.2±2.4	4.0±3.3^*^
%HF_5min_ (%)	0.1±0.4	0.2±0.6
*NREM phase*		
AHI (events/hr)	40.8±23.0^†^	28.0±29.9^†*^
Minimum SpO_2_ (%)	78.7±9.5^†^	83.3±7.5^†*^
Heart rate (/min)	61.4±8.1^†^	61.2±6.3^†^
HF (msec)	10.5±5.4^†^	10.9±5.9^†^
%HF_20sec_ (%)	30.3±16.6^†^	42.7±18.7^†*^
%HF_5min_ (%)	9.6±10.2^†^	20.8±16.3^†*^

HF: average instantaneous amplitude of the main HF peaks. ^†^ p<0.05 for comparisons of REM and NREM sleep in either gender. ^*^ p<0.02 compared to the males.

**Table 3:** Partial correlation coefficients for parasympathetic nerve function during REM and NREM sleep in males (M) and females (F) with OSA.

		REM			NREM		
		HF	%HF_20sec_	%HF_5min_	HF	%HF_20sec_	%HF_5min_
AHI	M	0.210^*^ (0.005)	–0.177^*^ (0.017)	–0.007 (0.923)	0.127 (0.087)	–0.518^*^ (0.000)	–0.461^*^ (0.000)
	F	0.192 (0.172)	–0.036 (0.799)	–0.128 (0.365)	–0.041 (0.774)	–0.407^*^ (0.003)	–0.268^*^ (0.040)
Age	M	–0.149^*^ (0.045)	0.009 (0.900)	0.041 (0.583)	–0.295^*^ (0.000)	–0.112 (0.132)	–0.128 (0.085)
	F	–0.220^*^ (0.048)	0.140 (0.324)	0.090 (0.528)	–0.357^*^ (0.009)	0.041 (0.771)	–0.128 (0.365)
Height	M	0.041 (0.583)	–0.005 (0.944)	–0.057 (0.445)	–0.031 (0.683)	0.004 (0.960)	–0.029 (0.699)
	F	0.137 (0.091)	–0.005 (0.969)	–0.067 (0.635)	0.189 (0.181)	0.040 (0.780)	–0.021 (0.880)
BMI	M	–0.187^*^ (0.012)	–0.079 (0.293)	–0.060 (0.423)	–0.174^*^ (0.019)	–0.013 (0.864)	–0.049 (0.515)
	F	–0.180 (0.201)	–0.369^*^ (0.007)	–0.097 (0.689)	–0.236^*^ (0.043)	–0.083 (0.560)	–0.123 (0.385)
Pack– years	M	0.000 (0.997)	–0.101 (0.454)	–0.163 (0.230)	–0.058 (0.669)	0.051 (0.711)	–0.063 (0.643)
	F	–0.068 (0.456)	0.042 (0.897)	–0.059 (0.579)	0.011 (0.974)	0.121 (0.394)	0.061 (0.510)

Pack-years: life-long cigarette consumption. AHI: AHI during either REM or NREM sleep. HF: average instantaneous amplitudes of the main HF peaks. The p-value of each partial correlation coefficient is indicated in parentheses. ^*^ Partial correlation coefficient with statistical significance (p<0.05).

In addition to the aggravating effect on the stability of the parasympathetic nerve function, apneas enhanced the extent of the parasympathetic-nerve-inducing cardiac modulation, but this was observed only during REM in male OSA (Table 3 and Fig. 2). It is not clear why the apnea-related enhancing effect of the parasympathetic-nerve-inducing cardiac modulation did not exist during REM in female OSA and during NREM in both male and female OSA. The findings observed in the present study are partly in accordance with those reported by Park et al. [31], who showed that AHI was importantly correlated with time-domain indices of HRV reflecting parasympathetic nerve function in male OSA. However, Park et al. [31] did not analyze the influence of AHI on parasympathetic nerve function in female OSA. Furthermore, they did not estimate the important role of AHI in eliciting the instability of the parasympathetic nerve function.

### Importance of aging for modifying parasympathetic nerve function

Differing from AHI, aging exerted no influence on the parasympathetic nerve stability. However, it acted as a universal factor in the diminishment of the parasympathetic-nerve-associated modulatory effect on the cardiac sinus node irrespective of sleep stage and gender; i.e., little gender-specific difference in the effect of aging on the parasympathetic nerve system (Table 3 and Fig. 4). The importance of aging for blunting the cardiac parasympathetic-nerve modulation in subjects with normal respiration was argued by many authors [9]–[15], attaining a reliable conclusion that the cardiac modulation by the parasympathetic nerve system declined with age. Reardon and Malik [12] considered that the age-dependent reduction in the parasympathetic-nerve-associated cardiac modulation was ascribed to the decreased responsiveness of the autonomic nerve system to exter­nal stimuli with age. Based on the time-domain analysis of HRV, Jensen-Urstad et al. [14] showed that the association between age and time-domain variables reflecting the parasympathetic-nerve-related cardiac modulation was somewhat weak in healthy females in comparison with that in males; i.e., the gender-specific difference in the effect of age on the diminution of the parasympathetic-nerve-associated modulatory effect on the cardiac sinus node. Although the methodological difference exits between the two studies, the integration of the findings reported by Jensen-Urstad et al. [14] and us may suggest that the age-dependent diminishment of the parasympathetic-nerve-associated cardiac modulation is weak in healthy women but exaggerated in women with OSA. The time-domain analysis for HRV conducted by Song et al, [23] demonstrated that the HRV indices corresponding to the parasympathetic-nerve-associated cardiac modulation sensitively responded to age in OSA patients, being highly consistent with the tendency observed in the present study.

### Importance of BMI for modifying parasympathetic nerve function

BMI, but not height, significantly blunted the parasympathetic-nerve-eliciting cardiac modulation during REM in males and during NREM in both males and females (Table 3 and Fig. 5). Furthermore, BMI made the parasympathetic nerve function considerably unstable during REM sleep in females but not in males (Table 3 and Fig. 5). These facts suggest that, contrary to the effect of age, BMI modifies the parasympathetic nerve function in a gender-specific manner. The importance of BMI or obesity for reducing the parasympathetic-nerve-eliciting cardiac modulation in subjects without apneic events was addressed by several authors [16]–[20], resulting in that BMI or obesity should be taken as a factor depressing the parasympathetic nerve activity. Nagai et al. [32] and Molfino et al. [18] conceived that reduction of parasympathetic nerve activity as body size increased might represent a defensive mechanism against fat deposition. Qualitatively the same trend concerning the inhibitory effect of BMI or obesity on the parasympathetic nerve activity was reported in the patients with myocardial infarction [17] and those with OSA [33]. Although the findings obtained in the present study for OSA patients are evidently consistent with those reported by other authors, we elucidated the effect of BMI on the parasympathetic nerve system in a more detailed fashion; i.e., the dependence of the BMI effect on gender and sleep stage as well as the separate effect of BMI on the parasympathetic-nerve-eliciting cardiac modulation and on the parasympathetic nerve stability.

### Effect of smoking habit on modification of parasympathetic nerve function

Although Barutcu et al. [21] revealed that cigarette smoking had a significant impact on depressing the parasympathetic-nerve-associated cardiac modulation in heavy smokers, Zhang et al. [22] did not obtain the evidence for reliably supporting the decreased parasympathetic-nerve-associated cardiac modulation when acutely exposing cigarette smoke to construction workers. On the other side, there was no authentic study shedding light on the significant role of smoking habit in modifying the parasympathetic nerve function in OSA patients. We found no influence of life-long cigarette consumption on the parasympathetic nerve function including its cardiac modulatory effect and stability (Table 3), being seemingly agreed with the findings reported by Zhang et al. [22].

### Clinical implication

Since the sympathetic nerve was confirmed to be conspicuously activated during the period with REM as well as NREM in OSA patients [34], [35], the age- and/or BMI-associated depression of the parasympathetic-nerve-eliciting cardiac modulation during REM and NREM in OSA patients would certainly augment the imbalance between sympathetic and parasympathetic systems especially in old and obese patients with OSA, leading to a high vulnerability to cardiovascular diseases in these patients. Since there was no reliable method for monitoring the transitional change in the autonomic nerve discharge in a continuous manner, the pathophysiological implication on the instability of the parasympathetic nerve system in subjects with and without apneas was never analyzed, In the present study, however, we were able to estimate the stability state of the parasympathetic nerve discharge using the instantaneous time-frequency analysis, leading to the conclusion that the parasympathetic nerve stability in patients with severe OSA would be substantially impaired. The enhanced instability of the parasympathetic nerve system may also augment the sympathetic-parasympathetic imbalance in patients having severe OSA.

### Study limitations

We should acknowledge that there is some possibility that the OSA patients recruited for the present analysis are contaminated with “selection bias” upon their visit to our Sleep Center. In fact, the average age of our patients is older, while the ratio of males and females is higher, than those investigated for OSA patients in general population. We consider that these propensities may be ascribed, in part, to the fact that our Sleep Center mainly targets the OSA patients who have severe apneas that have been left untreated for a long time, shifting the patient’s age at a time point when he or she is referred to our Center to an advanced direction.

It should be noted that we cannot answer the question of whether the extent of the age- and/or BMI-dependent inhibition of parasympathetic nerve discharge quantitatively differs between OSA patients and subjects with no apneas, because we did not examine the impact of age and BMI on the parasympathetic-nerve-evoked modulatory effect in non-apneic subjects. This is because the number of the subjects with no apneic episodes was very limited (n = 18) among the subjects referred to our Center. It was difficult, therefore, to have a reliable statistical evaluation with regard to the influence of various confounding factors on parasympathetic nerve function during sleep in subjects with no apneic events.

It should be acknowledged that the influence of the age-related restriction on the left ventricular function (i.e., the decrease in the left ventricular ejection fraction with age) was not excluded in the present study, because we did not evaluate the patient’s cardiac state by echocardiography.

Although we observed the gender- or sleep-stage-specific difference in the impact of AHI or BMI on the parasympathetic nerve function, we have no trustworthy explanation for this phenomenon at the present time. In addition, we found no influence of cigarette consumption on the parasympathetic nerve function in female OSA, this is not conclusive because 71% of the female participants are never smoked (Table 1). Further studies are actually required to fully elucidate the significance of the matters indicated above for modifying the parasympathetic nerve function in OSA patients.

In conclusion, the extent of the parasympathetic-nerve-eliciting cardiac modulation during REM and NREM in OSA patients is universally inhibited by aging irrespective of the gender; i.e., no gender-specific contribution of age to the parasympathetic-nerve eliciting cardiac modulation. BMI has the same effect as age but its effect is lacking during REM in female OSA. Apneas augment the extent of the parasympathetic-nerve-eliciting cardiac modulation but it is observed only in REM for male OSA. Concerning the stability of the parasympathetic nerve system, the apnea acts as the key factor for making it unstable in patients with OSA. The exception is recognized during REM in female OSA, for which BMI plays a significant role in inducing the parasympathetic nerve instability. These findings suggest that the effects of apneas and BMI on the instability of the parasympathetic nerve system are gender-specific. Cigarette consumption has no impact on any parasympathetic nerve function.
